# The National Insurance Bill

**Published:** 1911-07-01

**Authors:** 


### THE NATIONAL INSURANCE BILL.

### PROPOSED AMENDMENTS.

Notices of amendments to the National Insurance Bill already cover something like sixty pages and the number is increasing daily. The amendments to Clause 14, which have been drafted by the executive sub-committee of the State Sickness Insurance Committee of the British Medical Association, are in terms similar to those of which notice has been given by Dr. Addison and Mr. Harold Smith, the purport of which was explained in The Hospital last week. An amendment to Clause 8 drafted by the sub-committee is intended to limit the medical benefit to persons whose incomes do not exceed an average of £2 per week. A draft amendment to Clause 13 proposes that medical and maternity benefit shall, like the sanatorium benefit, in all cases be administered by the local Health Committees, and the intention of amendments to other clauses is to transfer the administration of these benefits to the same authorities. The amendments designed to secure adequate medical representation in the administration of the proposed scheme provided that the Board of Commissioners and the Advisory Committee shall include registered medical practitioners who have had personal experience of general practice among the classes from which the insured are drawn, and that in the case of local Health Committees, members to the number of at least one-fourth of the whole committee shall be appointed from among registered medical practitioners resident in the county or county borough.

### Local Medical Committees.

It will also be proposed to insert a new clause providing for the formation of local medical committees to be consulted by the Health Committees on all matters affecting the arrangements with medical practitioners. A further amendment drafted on behalf of the British Medical Association proposes to delete Clause 17, which empowers friendly societies to subscribe to hospitals and other charitable institutions. Sir Samuel Scott has given notice of an amendment, the purpose of which is to allow a mother in receipt of maternity benefit to decide whether she shall be attended by a registered medical practitioner or by a duly certified midwife. As to the supply of drugs and medicines under the proposed scheme, amendments have been put down with the object of securing that arrangements for this purpose shall be made with duly registered chemists and druggists.

### Some Questions.

A number of questions relating to the medical service under the proposed scheme have been asked in the House of Commons. The object of several of these was to ascertain whether it was not advisable to exclude the inmates of orphanages, voluntary homes, penitentiaries and similar institutions from the Bill or to suspend payments and benefits in respect of such inmates during the period of residence. The Chancellor's reply to these questions was to the effect that the objection to excluding such persons is that they might come into insurance later in life and bring with them heavy risks to be borne by the whole body of contributors; he has, however, some hope of arriving at a reasonable settlement of the difficulty. Asked whether he was aware that the medical profession is practically unanimous in asking for a wage limit beyond which free medical benefit should not be given, the Chancellor, through Mr. Wedgwood Benn, replied that it was impossible to argue such a complicated matter in reply to a question. Mr. Joynson-Hicks asked whether the Government had yet considered the point in the management of consumption sanatoria regarding patients who prove to be incapable of recovering, and in reply it was stated that the administration of sanatorium benefit would be in the hands of the local committees.

